# Special Care Dentistry for People in Asia-Pacific Region

**DOI:** 10.1016/j.identj.2025.100859

**Published:** 2025-09-22

**Authors:** Gustavo Fabián Molina, Chun-Pin Lin, Olabode Ijarogbe, Elham Kateeb, Hiroshi Ogawa, Syed Mahmood Shah, Fernando Fernandez, Ting-Chen Chen, Ting-Yi Renn, Chun Hung Chu, Wei-Jen Chang

**Affiliations:** a School of Dentistry, Facultad de Ciencias de la Salud, Universidad Católica de Córdoba, Argentina; b The Faculty of Dentistry, Universidad Nacional de Córdoba, Argentina; c Department of Dentistry, National Taiwan University Hospital, Taipei, Taiwan; d Graduate Institute of Clinical Dentistry, School of Dentistry, National Taiwan University, Taipei, Taiwan; e Asia Pacific Dental Federation, Manilla, Philippine; f Department of Restorative Dentistry, Faculty of Dental Sciences, College of Medicine, University of Lagos, Lagos, Nigeria; g Oral Health Research and Promotion Unit, Al-Quds University, Jerusalem, State of Palestine; h Division of Preventive Dentistry, Department of Oral Health Science, Graduate School of Medical and Dental Sciences, Niigata University, Niigata, Japan; i Department of Orthodontics, Muhammad Dental College, Mirpurkhas, Sindh, Pakistan; j Department of Oral Health, Ministry of Health and Welfare, Taiwan; k School of Dentistry, College of Oral Medicine, Taipei Medical University, Taipei, Taiwan; l Division of Restorative Dental Sciences, Faculty of Dentistry, The University of Hong Kong, Hong Kong, China; m Dental Department, Shuang-Ho Hospital, Taipei Medical University, New Taipei City, Taiwan

**Keywords:** Special dental care, Asia-Pacific, Caries, Oral health promotion

## Abstract

The Asia Pacific Dental Federation hosted the Asia Pacific Dental Congress 2024 and convened public health experts to develop recommendations for Special Care Dentistry (SCD) for Asian countries. Many countries strive to promote oral health and ensure equal access to oral care for individuals with disabilities, but the implementation of SCD varies significantly. Leading the way in SCD education, research and well-organised services are countries such as Japan, Malaysia and Korea. Meanwhile, Hong Kong, Singapore, Taiwan and Thailand are introducing changes to their SCD educational and health systems. Publications from Pakistan, Indonesia and the Philippines indicate a growing interest in these issues, while informal reports from peers suggest a similar trend in Vietnam. Essential components of oral care for individuals with disabilities include accessibility, education, prevention, awareness, specialised dental care, and collaboration among stakeholders. The expert panel has put forth a series of recommendations as follows: (1) ensuring barrier-free access to dental care facilities for individuals with physical disabilities, (2) enhancing oral health promotion and oral care provision for people with disabilities, (3) promoting collaboration among health policymakers and institutions in Asian countries and region, (4) implementing mandatory education and training on SCD in universities and professional institutions, and (5) emphasising the need for a multidisciplinary approach to provide holistic care and bridge the gap between health allied professions. These recommendations aim to address inequalities and discrepancies in access to oral care, aligning with the principles outlined in the United Nations Convention of Rights for People with Disabilities.

## Introduction

Over one billion people live with some form of disabilities worldwide, and that number is dramatically increasing due to demographic trends and increases in chronic health conditions, among other causes.^1^ Oral health policy for this population should be defined and taken into action with effective strategies based on available evidence, integrated with general health policy and supported by committed oral health–related institutions. This is very crucial because literature documents that persons with disabilities continue to face considerable barriers when accessing health care services, which negatively affects their chances of achieving their highest attainable standard of health. Although there is growing evidence of interventions targeting unequal access to health care services, they remain too few and sparse to meet the populations' needs. Profound systemic changes and action-oriented strategies are warranted to promote equitable access to care for persons with disabilities and advance global health priorities.^2^

According to data retrieved from public documents, in Japan, Taiwan, South Korea, Hong Kong, Thailand, Malaysia, Singapore, Indonesia, China and the Philippines, the oral health policy for people with disabilities aims to ensure equitable access to dental care and promote oral health for individuals with disabilities.^3^, ^4^, ^5^, ^6^, ^7^, ^8^, ^9^, ^10^ Anecdotal information from Vietnam and Cambodia shows the same trends than in their peer countries in the region. The policy focuses on the following key aspects: accessibility, education/prevention/awareness activities, specialised dental care and collaboration with policymakers, other stakeholders and the health care multidisciplinary team.

Countries such as Pakistan, India, Afghanistan, Bangladesh, Myanmar and Nepal add billions of people living in the Asia-Pacific Region with a set of demographic characteristics and particular health-related concerns.^11^^,^^12^ Nevertheless, oral health status among people with disabilities worldwide has been reported to be poorer compared to the general population due to unmet treatment needs, irrespective of geographical location, socio-economic level or availability of services.^13^, ^14^, ^15^, ^16^, ^17^, ^18^

Efforts are made to improve accessibility to dental care facilities by ensuring barrier-free access for people with physical disabilities. This includes the provision of wheelchair ramps and elevators in dental clinics and hospitals, accessible dental equipment, and facilities that cater to the specific needs of individuals with disabilities. Dental facilities are also encouraged to have appropriate dental equipment and resources to accommodate the needs of individuals with disabilities.

The need for improved accessibility is not just for those with physical impairment but also for those with other types of disabilities such as communication difficulties or visual impairment. People with such challenges may need aiding tools which would help them to get the required information or to get to know their options. Moreover, an appropriate atmosphere may help to reduce anxiety for those with dental fear or behavioural problems.

Regarding education, public awareness campaigns and educational programs have been conducted in most of the Asia-Pacific region to promote oral health and hygiene among people with disabilities.^19^^,^^20^ These initiatives aimed to increase understanding of the importance of oral health and provide information on appropriate oral care practices. However, the impact of such initiatives remains uncertain.

Current policies in countries such as Japan, Korea, Taiwan and Malaysia emphasise the provision of specialised dental care services for individuals with disabilities. This includes training dental professionals in the management of patients with special needs as well as establishing specialised dental clinics equipped to handle the unique requirements of patients with disabilities.^3^, ^4^, ^5^, ^6^, ^7^, ^8^, ^9^

From an academic point of view, although core contents related to special care dentistry (SCD) can be identified in most of the undergraduate curricula throughout Asian dental schools, it is unlikely to confirm that students receive standardised clinical training for managing patients with disabilities. This issue has been discussed in the Southeast Asian Association for Dental Education among participants from the member associations, at the 2022 (Siem Reap, Cambodia) and 2023 (Singapore) annual meetings,^19^^,^^20^ leading to a strategic collaborative plan for the implementation of SCD in the undergraduate curricula based on best practices documented in the world.^20^, ^21^, ^22^

The provision of oral health care for people with disabilities is, perhaps, the aspect where disparities become more evident. Such variations depend not only on each country’s dominant health system but also on the diversity of contexts within each country or region. However, irrespective of these determinants, preventive strategies and basic packages for oral care are a matter of concern for the public sector in every Asian country because resources for special needs oral health care (SNOH) are noticeably limited in this region.^8^

The challenges are to strengthen these basic services with quality assurance as well as to enhance the provision of quality treatment in complex cases, enhancing the pathways for referrals, thus reducing inequalities in access to care for people with disabilities in more disadvantaged regions.^9^^,^^10^

These policies also encourage collaboration between dental professionals, caregivers and relevant organisations to ensure comprehensive oral health care for individuals with disabilities. This cooperation helps to address any challenges and provide appropriate support to individuals with disabilities during dental visits.

It's important to note that policies and practices may vary across different regions and health care settings. Therefore, it is advisable to consult local dental clinics or relevant authorities for specific information regarding oral health policies for people with disabilities within each country or region to get an insight on the situation and to pilot the feasibility of any general policy applied to these particular areas.

The Asia Pacific Dental Federation (APDF) intends these recommendations for improving access to oral health care of people with disabilities in Asian countries and regions to align with the principles outlined in the United Nations Convention of Rights for People with Disabilities.^23^ These recommendations would only be feasible with the engagement and advocacy of all stakeholders involved in the oral health of this population.

## Methods

An Asia Pacific Dental Congress (APDC) SCD working group was formed by a panel of health experts who reviewed the literature and retrieved anecdotal information from experts/peers and developed SCD recommendations for different stakeholders in Asian countries and regions.

The search strategy used an initial screening of governmental documents (administrative, legislative or policy statements) aided by artificial intelligence (e.g. ChatGPT), followed by a thorough search on electronic databases (The Cochrane Library database and PubMed) and anecdotal information gathered from Asian peers with expertise in SCD.

**Fig. 1:**
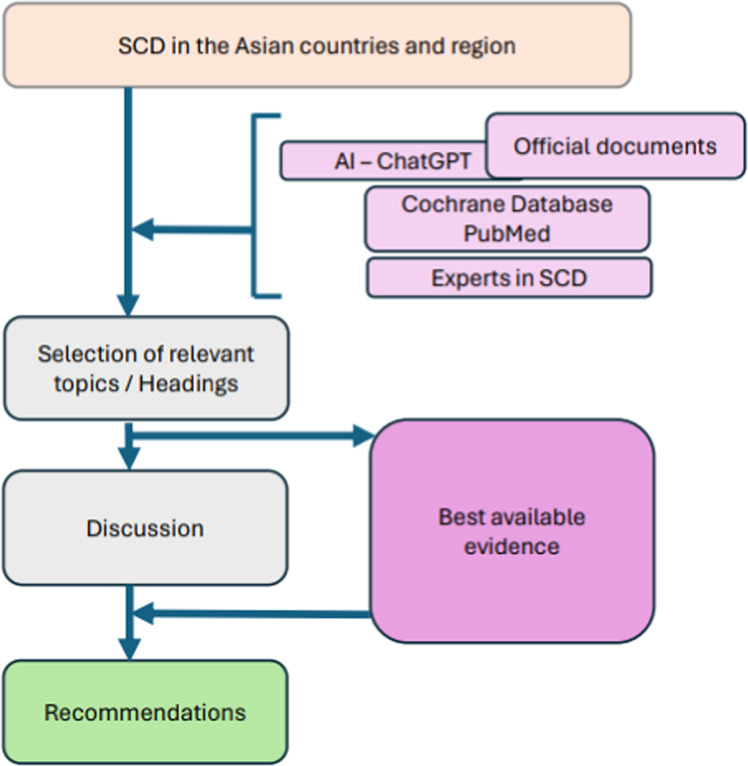
Flow chart for the development of recommendations on special care dentistry in Asian countries and region.

Information retrieved from different sources was discussed among the members of the APDC SCD working group, and relevant topics were identified for expanding the discussion and producing recommendations based on best evidence. Each recommendation begins with a heading for the correspondent relevant topic, followed by suggested actions with their respective supporting references. A flow chart for the development of these recommendations is displayed in Figure 1.

## Recommendations and actions

*Efforts should be made to improve accessibility to dental care facilities by ensuring barrier-free access for people with physical and intellectual disabilities.*

Persons with disabilities may face a range of barriers when they seek oral health care. The wide scope of impairment types may expose them to certain types of barriers for accessing services that also vary in different geographic/cultural settings and different policy environments.

The first barrier that can be mentioned is the lack of accessible transport, or lack of nearby facilities, which may limit people with disabilities’ access to health services.^24^

Among different barriers that are listed by the users of dental services for people with disabilities, architectural boundaries are one of the main difficulties that need to be sorted out.^25^ Obstacles can make it difficult not only to enter the facility but also to move through elevators or doorways into treatment rooms or use the bathrooms. Having accessible/adaptable furniture or equipment is also an important consideration.^24^^,^^26^

To enable access to dental care facilities, it is essential to ensure barrier-free access for people with restricted motricity or sensory limitations as well as for the ageing population.^25^^,^^27^^,^^28^ This includes the provision of wheelchair ramps and/or elevators, accessible dental equipment and facilities that cater to the specific needs of individuals with disabilities in dental clinics and hospitals. It would be optimal to have available waiting rooms with easy access to wheelchairs and with appropriate spacing.^29^^,^^30^ Dental facilities are also encouraged to have appropriate dental equipment and resources to accommodate the needs of individuals with physical impairment or bariatric problems.^31^

Other types of barriers may be less well recognised and understood. For example, people who are deaf or have other communication difficulties (e.g. cognitive or psychosocial impairments) may miss out on information or being informed about their options if there is a lack of sign language or other adapted communication tools.

People with visual impairment may not be able to read certain types of handwriting, instructions regarding medication or even a screen with announcements.^24^ Therefore, clear signage in easy-to-read lettering or Braille language or loudspeakers for announcements would be desirable, both in waiting and treatment rooms.

Lastly, individuals living with autistic spectrum disorder may also need adaptations of the dental environment to make it more accessible and inclusive, such as the use of certain colours, scents or music.^32^

### Prevention

*Oral health promotion as well as the provision of oral health care to people with disabilities should be encouraged to aim for equal treatment outcomes in comparison to the general population.*

Public awareness campaigns and educational programs are conducted to promote oral health and hygiene among people with disabilities. These initiatives aim to increase understanding of the importance of oral health and provide information on appropriate oral care practices.

Strengthening oral health promotion is a major aim stated in strategic planning to spearhead improvements in the oral health of the general population as well as among people with special health care needs. The actions most likely to be included are to incorporate oral health messages and services available for the special groups into the overall oral health promotion campaigns, to organise oral health campaigns at institutions/schools for teachers, carers, minders and parents of children with special needs, and to introduce oral health care aids specific to disability.^33^

A wider approach to this issue would be to seek strategic alliances with oral health care brands. This collaboration may help to align common aims among all participants involved that may eventually benefit consumers by improving access to preventive products on a larger scale.^34^ Nevertheless, ethical concerns for potential conflict of interests should be examined beforehand to avoid compromising the nobility of the ultimate goals.

### Provision of care

Equity in health implies that people in equal need should have equal access, equal treatment, and equal treatment outcomes within the health system. However, massive inequality in the quantity and quality of dental treatment provided for people with disabilities is still a matter of concern.^35^ The literature confirms significant unmet need, and that dental extraction is the most prevalent treatment option.^29^^,^^36^

The basic right to an equivalent level of oral health care should be granted. Therefore, SCD should not be perceived as the discipline of compromise where ‘better than nothing’ is the bottom line.^29^^,^^36^ For the provision of quality care, a first step should be the revision of best practices that are currently carried out in reference centres in Asia to serve as the basis for future guidelines for the region.

The best evidence should be collected, exchanged and discussed in scientific meetings. This information should be shared with the patients/carers so they could choose and decide the most suitable treatment amongst all available options based on scientific evidence.

*Collaboration amongst health policymakers and institutions in Asian countries should be encouraged to promote equal access to and provision of quality oral health care for people with disabilities.*

Access to oral health care for people with disabilities is determined by the type of health system in each country and the local context. To enable access, the health service must meet the needs of the user, be available and be appropriate to the population.^37^ The three priorities of health care delivery—cost, quality and access—need not be at odds with each other. Greater use of health care facilities due to lower costs and shorter distances to travel will only lead to better health outcomes if quality of care is guaranteed. Therefore, it would be desirable to make high-quality health care services affordable and accessible to effectively implement universal health coverage.^38^

The policy encourages collaboration between dental professionals, caregivers and relevant organisations to ensure comprehensive oral health care for individuals with disabilities. This cooperation should help to address any challenges and provide appropriate support to individuals with disabilities during dental visits.

It is important to note that policies and practices may vary across different regions and health care settings. The development of local programs and policies have been suggested to address oral health access problems in each region, pertaining to (1) improving workforce supply and distribution, (2) education reform and increased public accountability, (3) practice reform and (4) increased data collection and research.^39^ Similarly, other sources have identified areas for the development of collaborative programs within the Asian region. Suggested ways to collaborate were categorised into the following areas: engagement, advocacy, policies, specialty and upskilling.^40^

In 2018, the Taiwan Ministry of Health and Welfare launched the New South Bound Special Needs Patients Oral Care Project, executed by Chung Shan Medical University, aiming to extend Taiwan’s model of SNOH to southeast Asian countries. This project has engaged 4 countries and 6 universities so far, and have trained a total of 245 dentists, dental assistants or support staff, including 82 memorandums of understanding (MOUs) for medical health care agreements and 15 MOUs for cooperation agreements that were officially signed to establish a network for the referral of special-needs oral health care in the region.^8^

In parallel with these endeavours, it is important to receive continuous feedback and thus ensure a bidirectional flow of information: on one hand, it is advisable to consult local dental clinics or relevant authorities for specific information regarding oral health policies for people with disabilities to understand the diversity of areas in these countries; on the other hand, utilisation of services strongly depends on demographic and socio-economic factors. Understanding these factors could help to formulate effective interventions to improve oral health care utilisation.^41^

*Education and training at undergraduate and postgraduate level will be promoted, including special care dentistry as mandatory in dental curriculum.*

An estimated 16% of the global population experience significant limitations in functioning or suffer from severe disability, and this number is growing because of an increase in noncommunicable diseases and people living longer.^1^ Consequently, it is no longer possible to consider SCD as a discipline for a minority, to be taught only at the postgraduate level. Despite the increased interest in SCD, education including classroom teaching and practical teaching is still insufficient and needs more development.^42^

People with disabilities are more likely to develop oral disease due to obstacles that prevent them from obtaining adequate personal and professional oral health.^43^ They tend to choose treatments and services full of empathy, professionalism and compassion, emphasising the need for enhanced intervention to provide preventive, curative and long-term oral health care.^44^

Dentists’ awareness and positive attitudes toward people with disabilities can improve oral health services.^45^ Therefore, new graduates in all dental disciplines must qualify with the appropriate skills, behaviours and attitudes to serve all members of the wider community.

Collaboration amongst dental schools of the Asian countries should be sought to reduce asymmetries in dental education regarding SCD. As mentioned in the Introduction, a proposal was presented at the Southeast Asian Association for Dental Education meetings in 2022 and 2023 to develop a plan for the implementation of SCD in the undergraduate curricula among the associated dental schools and faculties.^19^^,^^20^ The proposal includes the following sequence of actions:

1. To select a task force of teachers from each school/faculty that will lead on the implementation of SCD.
2. To review each school/faculty curriculum learning issues and learning outcomes and mark all those that match with the International Association of Disability and Oral Health undergraduate learning outcomes.
3. To list potential activities that may help to cover those learning outcomes from the International Association of Disability and Oral Health curriculum that do not have correspondence in each dental school/faculty curriculum.
4. To calibrate the task forces, selected teachers will get together to (1) define the contents that will be mandatory for the students to access and the sources that should be consulted by the students, (2) calibrate on the use of a case mix tool to define the complexity of care and decide what patients should be examined and treated at the undergraduate level, and (3) prepare a template of a portfolio for the students to achieve the missing competences that had been identified.

To achieve these aims, each dental school/faculty should guarantee sufficient clinical exposure to develop the knowledge, skills and attitudes that are expected as learning outcomes. A common e-platform was suggested to upload the portfolios, following ethical guidelines to safeguard the privacy of the information and the anonymity of the patients.

At a postgraduate level, available courses should be offered through a network of dental schools and associations, with CE eligibility for accreditation. It would be desirable that such courses include a multidisciplinary approach to promote the exchange of knowledge and skills while carrying out the training of oral health allied professionals, encouraging teamwork.

*Multidisciplinary approach should be emphasised to provide holistic care, bridging the gap between all health allied professions.*

Participation of health allied professionals in a multidisciplinary team has proven to be an effective strategy to tackle oral health problems that are not restricted to the oral cavity and have an impact on general health or systemic problems that may influence oral health.^46^

This holistic approach constitutes the essence of the WHO International Classification of Functioning, Health, and Disability (WHO/ICF) and provides a suitable framework for the development of multidisciplinary preventive and/or therapeutic programs.^47^, ^48^, ^49^

Dentists should be encouraged to engage in scientific meetings organised by other professionals of the multidisciplinary team, and vice versa. This exchange may enable new perspectives to approach more complex problems or those old problems that have never found sustainable solutions.

The production of scientific evidence should be shared in multidisciplinary forums or platforms such as journals or web-based resources for knowledge exchange.

To favour the development of such frameworks, policies, health systems and institutions should be aligned to the aim of a comprehensive approach and support these endeavours.

*A universal criterion to define the complexity of care should be developed (or adopted, if available) for enhancing pathways of care.*

In the past few decades, major technical advances in dentistry have led to improved quality of dental treatment and improved ability to maintain oral function and aesthetics over the lifespan. However, there are still gross and unfair inequalities in terms of the quantity and quality of dental treatment provided to people with disabilities.^30^^,^^35^

There is evidence that most but not all dental needs could be met in primary dental care settings.^49^ The problem for oral health care providers and governments is how to identify and select those who are best managed in the primary dental care setting and to decipher who needs additional specialist skills and adjuncts to receive and tolerate dental care.

Furthermore, there is a need to ensure that such dental care is optimised and personalised to the patient’s specific dental needs, considering that the scope of disability extends to a wide range of medical conditions. Although there is evidence supporting best practices for preventive and/or therapeutic strategies to alleviate disease levels in this population in Asian countries and globally, these have been systematised using the “medical model,” under the heading of a medical condition or diagnosis, which often leads to stigmatising people with disabilities.

Some countries have attempted to respond to this problem by developing “case mix” tools to describe the complexity of the management of patients with special needs.^48^, ^50^, ^51^ To date, these local tools have been produced to justify additional financial resources or for service commissioning. A validated, universal tool could have a much wider purpose. Such a tool could effectively identify the adaptations required for dental service provision and for individual patient care; it could provide clear epidemiological data for service policy and planning; it could justify resource deployment for education and necessary adjuncts (such as sedation and general anaesthesia services); it could provide practical guidelines for the orientation and referral of patients between primary, secondary and tertiary dental services where available; and it could thus lead to more efficient and equitable distribution of oral health services and outcomes for people with special care dental needs. Moreover, it could have a significance to research because it might be used as a standardised means of describing the care needs of participants in clinical research and to ensure inclusion of persons from disability or disadvantaged groups in research protocols.^52^ Reproducibility of research will be improved and the power of research in rare diseases increased, as well as providing a tool for networking.

*Public and private services should have the availability of different resources for behaviour support.*

A combination of factors such as fear of potential comorbidities associated with interventions under general anaesthesia (GA) or dental sedation (DS), along with the lack of these resources, contributes to increased efforts to grow awareness among the families of patients with disabilities about the importance of focusing on prevention of the disease to avoid complex treatment solutions. Furthermore, the use of minimally invasive approaches has also been demonstrated as suitable and effective in reducing the need for referral to GA or DS.^53^

GA and DS, as well as other resources for behaviour support, are used as an adjunct to dental treatment for people with disabilities. The frequency of GA use varies widely, from 0.5%-20% up to as high as 70%.^54^^,^^55^ This variation can be explained by population diversity and the lack of uniformity in measuring the need for GA, although it has been observed that the need for these procedures increases with the complexity of care. Service design, cultural norms and clinical judgements can coalesce in the decision-making process for the referral of dental treatment under some type of GA or DS. Moreover, limited availability of such services in developing regions may add a substantial barrier to the provision of quality care, neglecting optimal selection of dental treatment and, in some cases, not even covering an emergency phase.

The availability of advance behaviour support adjuncts is certainly tied to the ability of each country or regional administration to invest in technology and infrastructure. Nonetheless, there is a large list of strategies that dental teams ought to learn to offer new options where this availability seems to be restricted, because of either economic or organisational burden, at least until a brighter future of technology arrives.^56^

Table 1 summarises the recommendations and actions agreed upon following discussions within the working group, based on the existing evidence retrieved from international publications and consultations with regional experts on SCD. Notice that some recommendations should be approached by different stakeholders to achieve sustainable results.

**Table 1:** Recommendations for stakeholders on special care dentistry in the Asian countries and region

Actions
**Policymakers**
• *To improve accessibility to dental care facilities by ensuring barrier-free/friendly access for people with intellectual and/or physical disabilities* • *To promote oral health and to aim for oral health care with equal treatment outcomes* • *To increase public awareness of special care dentistry* • *To emphasise multidisciplinary approach for holistic care, bridging the gap between all health allied professions* • *To develop or adopt a universal criterion that can determine the complexity of care of patients with special health care needs* • *To make available different resources for behaviour support*
**International and national dental associations**
• *To encourage collaboration amongst health policymakers and institutions to promote equal access to quality oral health care for people with disabilities* • *To emphasise multidisciplinary approach for holistic care, bridging the gap between all health allied professions* • *To develop or adopt a universal criterion that can determine the complexity of care of patients with special health care needs* • *To advocate and claim for Persons with Disability’s rights supported by the United Nations Convention on the Rights of Persons with Disability*
**Individual oral health care professionals**
• *To emphasise multidisciplinary approach for holistic care, bridging the gap between all health allied professions* • *To make available different resources for behaviour support* • *To advocate and claim for Persons with Disability´s rights supported by the United Nations Convention on the Rights of Persons with Disability* • *To increase awareness among the families of patients with disability to focus on disease prevention*
**Academia**
• *To advocate and claim for Persons with Disability´s rights supported by the United Nations Convention on the Rights of Persons with Disability* • *To provide continuing professional education and specialty training on SCD to dental students and dentists* • *To emphasise multidisciplinary approach for holistic care, bridging the gap between all health allied professions*
**Parents or guardians**
• *To advocate and claim for Persons with Disability´s rights supported by the United Nations Convention on the Rights of Persons with Disability* • *To increase awareness among the families of patients with disability to focus on prevention of the disease to avoid complex treatment solutions*
**Educational institutions**
• *To emphasise multidisciplinary approach for holistic care, bridging the gap between all health allied professions*

## Conclusions

An increasing number of people in the Asia-Pacific region experience significant limitations in functioning or suffer from severe disability, and oral health play a significant role in their wellbeing. All stakeholders are responsible for improving access to oral care for people with disabilities with adaptations according to each particular context. The recommendations provide some insights on how to improve the oral health care policies, health care system and oral health care promotion and practice for this growing population.

## Conflict of interest

None disclosed.
